# A bibliometric and visualization analysis of research trends and hotspots on targeted therapy for breast cancer from 2003 to 2022

**DOI:** 10.3389/fonc.2024.1366900

**Published:** 2024-06-04

**Authors:** Deqi Wu, Chi Pan, Yangying Hu, Zhijie Shi, Yankun Zhou, Min Xiao

**Affiliations:** ^1^ Department of Thyroid and Breast Diagnosis and Treatment Center, Shulan (Hangzhou) Hospital, Shulan International Medical College, Zhejiang Shuren University, Hangzhou, China; ^2^ Department of Breast Surgery, The Second Affiliated Hospital, Zhejiang University School of Medicine, Hangzhou, China; ^3^ Zhejiang University School of Medicine, Hangzhou, China; ^4^ Department of Surgery, Shulan (Hangzhou) Hospital, Shulan International Medical College, Zhejiang Shuren University, Hangzhou, China

**Keywords:** breast cancer, targeted therapy, bibliometric analysis, Web of Science, trends, hotspots, VOSviewer, CiteSpace

## Abstract

**Background:**

Breast cancer is a significant public health issue, exhibiting the most pronounced occurrence and fatality rates among malignant neoplasms globally. Targeted therapy is a medical intervention that focuses on specific molecular markers. This study aims to investigate and evaluate the current research trends and directions in the field of targeted therapy for breast cancer using bibliometric analysis.

**Method:**

The Web of Science database was utilized to retrieve relevant articles published between 2003 and 2022. The VOSviewer software and Bibliometrix package in the R language were employed to conduct co-occurrence and clustering analyses of authors, countries, institutions, journals, references, and the CiteSpace tool was utilized for keyword burst detection.

**Results:**

A total of 2,258 articles were included and the annual number of publications increased rapidly. The most prolific country on this topic was the USA (n=898, 39.77%) and the University of Texas MD Anderson Cancer Center published most papers (n=93). Dennis J. Slamon and Gabriel N. Hortobagyi stood out in the field, with Dennis J. Slamon leading in terms of co-citations(n=653) and Gabriel N. Hortobagyi topping the list in terms of published articles(n=18). The most productive journal was Breast Cancer Research and Treatment and the most cited journal was Journal of Clinical Oncology. The clustering of keywords indicated that the primary focus of researches in the past two decades was on the development and clinical evaluation of tumor-targeted drugs associated with the epidermal growth factor receptor (EGFR) family signaling pathway, and explored mechanisms related to biological behavior of breast cancer. Keywords co-occurrence and burst analysis identified current research hotspots and potential research trends.

**Conclusion:**

This study employed bibliometric analysis to examine research on targeted therapy for breast cancer over a span of 20 years, and identified development trends of research and elucidated potential research trajectories in the domain of this topic. This study helps in the identification of prospective collaborators and partner institutions for researchers.

## 1 Introduction

Breast cancer is a prevalent public health concern, with the highest incidence and mortality rates among malignant tumors worldwide. According to the GLOBOCAN 2020 Global Cancer Statistics, there were an estimated 2.261 million new cases of breast cancer and 685,000 deaths from breast cancer worldwide in 2020 (1). In China, the incidence and mortality rates of female breast cancer have been steadily increasing, with approximately 303,000 new cases and 71,000 reported deaths in 2015 (2). Several risk factors contribute to the development of breast cancer, including genetic mutations, hormonal imbalances, lifestyle choices (e.g. smoking, obesity), and environmental factors (e.g. exposure to certain chemicals or radiation) (3). Breast cancer exhibits heterogeneity at the molecular level, with four subtypes differentiated based on distinct molecular characteristics (4). Treatment strategies vary accordingly for these molecular subtypes. As research continues to focus on the molecular characteristics of breast cancer, significant progress has been made in developing more effective and personalized treatment strategies for different subtypes. Tamoxifen is a selective estrogen receptor modulator (SERM) that has been used as a treatment for breast cancer for over 30 years. It is considered the first targeted therapy for breast cancer. Tamoxifen works by blocking the action of estrogen on breast cancer cells, which can help slow their growth and reduce the risk of recurrence. Its success has benefited millions of estrogen positive breast cancer patients (5). The introduction of the first targeted drug for human epidermal growth factor receptor 2 (HER2) positive breast cancer, trastuzumab, in 1998 marked a significant milestone in breast cancer treatment. Trastuzumab is a recombinant humanized monoclonal antibody that targets the HER2 protein, which is overexpressed in 15–20% of all breast cancer patients (6). By blocking the activity of this protein, trastuzumab can slow the growth of tumors and improve outcomes for patients with HER2-positive breast cancer. Since then, there have been numerous advances in targeted therapy for breast cancer, including tyrosine kinase inhibitors, poly (ADP-ribose) polymerase (PARP) inhibitors, vascular endothelial growth factor(VEGF) inhibitors, phosphoinositide 3-kinase(PI3K) inhibitors, immune checkpoint inhibitors, and cyclin-dependent kinase 4/6 (CDK4/6) inhibitors, and etc. (7, 8). Targeted therapy has improved the accuracy of anti-tumor activity and minimized toxicity to normal tissues. Thus in the era of precision medicine, targeted therapy plays an indispensable role in breast cancer treatment. With the increasing number of published studies and the extensive range of topics, it becomes imperative to conduct a quantitative analysis of targeted therapy for breast cancer. To gain a comprehensive understanding of the current research landscape and emerging trends in targeted breast cancer treatment, this study employed the Web of Science Core Collection database to scour and scrutinize literature spanning from 2003 to 2022.

Bibliometrics is a method of quantitative analysis and research that examines various aspects of academic literature, such as quantity, quality, distribution, influence, and more. It was first proposed by Alan Pritchard in 1969 with the goal of understanding the research status, development trend, and frontier dynamics of a specific field by analyzing the rules and characteristics of academic papers (9). The research objects of bibliometrics include literature volume, citation volume, author distribution, institutional distribution, keyword analysis, journal impact factor, and more. In this study, we aimed to conduct a bibliometric analysis of publications on targeted therapy for breast cancer over the past two decades to identify research hotspots, reveal development trends, and provide insights that can inform future research.

## 2 Materials and methods

### 2.1 Search for publications and data collection

**Figure 1:**
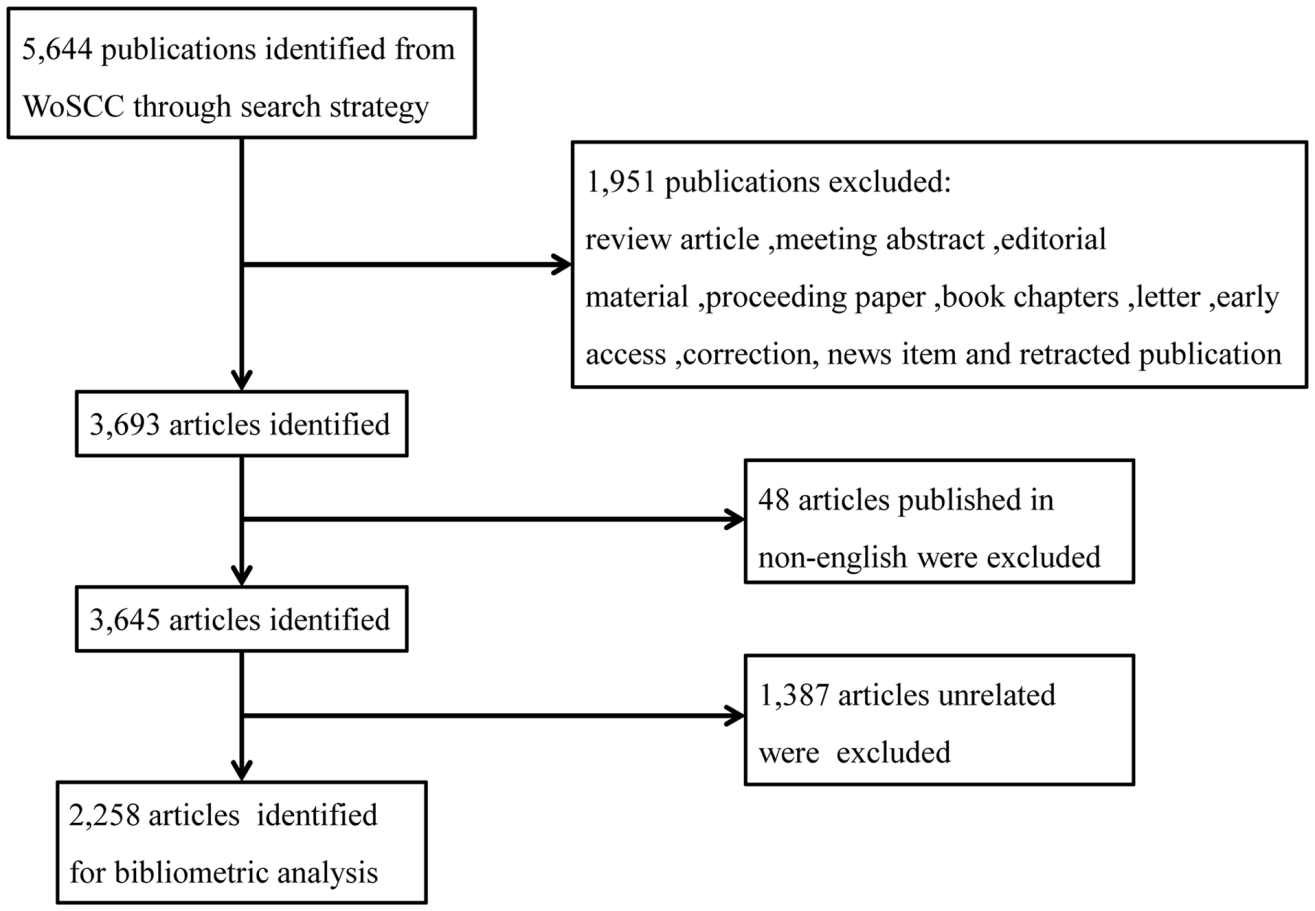
Flowchart of data collection and filtering.

The data were obtained from the Web of Science Core Collection (WoSCC) database, with the SCI-EXPANDED index selected. The data were sourced by the retrieval formula: 1# TI = (“Breast Neoplasm” OR “Breast Tumor” OR “Breast Carcinoma” OR “Breast Cancer” OR “Mammary Neoplasm” OR “Mammary Tumor” OR “Mammary Carcinoma” OR “Mammary Cancer”), 2# TS = (“Targeted Therap*” OR “Targeting Therap*” OR “Targeted Treatment” OR “Targeting Treatment”), 1# AND 2#. We refined the results by selecting the document types “article”, Timespan = 2003–2022 and the language “English”. A total of 3,645 articles were retrieved. Two of our authors screened and excluded search results based on the abstracts independently. Any differences in opinion were addressed through discussion, and 2,258 articles were retained finally. The data collection was completed on January 21^th^, 2023. The flowchart of data collection and filtering was shown in **Figure 1**.

### 2.2 Data analysis

Microsoft Office Excel 2010 software was used to depict the annual distribution of publications. We choose CiteSpace 6.1. R6, VOSviewer1.6.18 and R 4.3.0 for bibliometric analysis and create knowledge graphs. We imported the collected full records text information into VOSviewer and CiteSpace. VOSviewer software was used to extract and visualize the information of authors, co-cited authors, countries, institutions, journals, co-cited journals and co-cited references. We established collaborative networks of authors, countries and institutions. In the countries’ network visualization, we merged Taiwan into China and replaced England, Scotland, Northern Ireland and Wales with the United Kingdom. The bibliometrix package was installed in R and Biblioshiny software in the bibliometrix package was used to create countries’ collaboration network map (10). After cleaning the data by merging synonyms, keywords were clustered and co-occurred, and CiteSpace was used to analyze the keyword bursts of the literatures.

## 3 Results

### 3.1 Annual publication trends analysis

**Figure 2:**
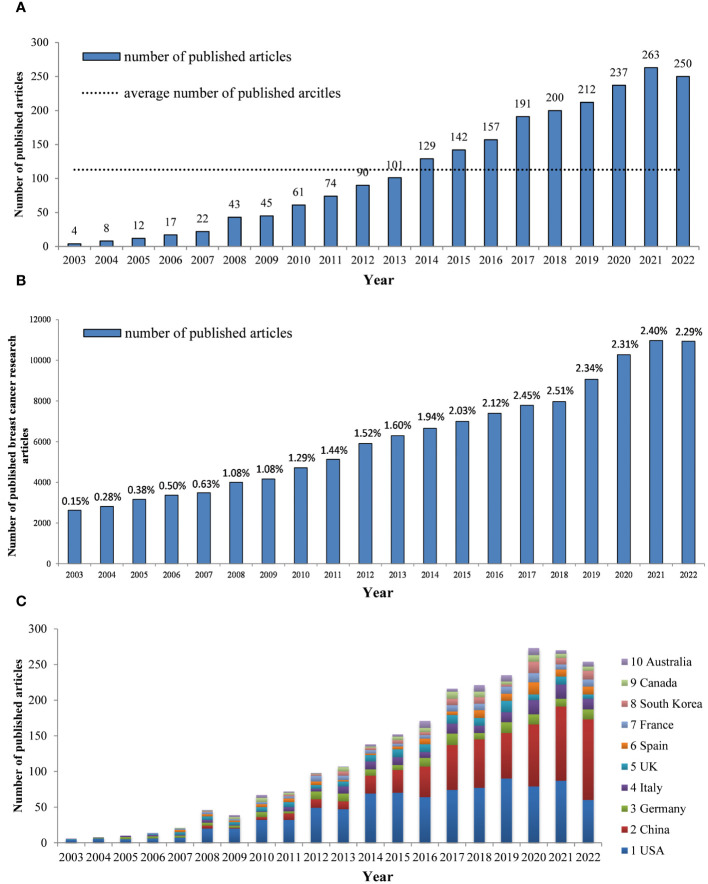
**(A)** The number of annual publications and growth trends of breast cancer targeted therapy (2003–2022) **(B)** The number of annual publications of breast cancer research and the proportion of targeted therapy in the breast cancer research field. (2003–2022) **(C)** Trends in the proportion of annual publications in top 10 countries.

A total of 2,258 papers related to breast cancer targeted therapy were included in this study, which were authored by 15,429 authors from 3,230 institutions across 85 countries. The papers were published in 559 journals and cited 67,694 references from 5,530 journals. The popularity of a research field can be gauged by the volume of publications. In the WoSCC database, we obtained a total of 2,258 publications related to the research area of breast cancer targeted therapy from 2003 to 2022, with an average annual publication volume of 113 and annual growth rate 24.07%. In 2003, only 4 articles were published. By 2014, the number of published articles had surpassed the average level. From 2003 to 2021, the annual number of published articles increased significantly, reaching 263 in 2021. In 2022, the number slightly decreased to 250 articles (**Figure 2A**). During the same timeframe, the annual number of articles published in the field of breast cancer research increased from 2,631 in 2003 to 10,937 in 2022, with an average annual growth rate of 7.38%. The share of literature concerning targeted therapy of breast cancer research saw a notable increase, which rose from 0.15% of the total research documents in 2003 to a peak of 2.51% in 2018. By 2022, this proportion slightly adjusted to 2.29%, yet still represented a substantial 15.27-fold increase from the original figure in 2003(**Figure 2B**). In conclusion, breast cancer targeted therapy research has received considerable attention and the number of published articles on this topic has been on the rise over the past two decades.

By analyzing the data from **Figure 2C**, we can identify trends in the proportion of annual publications in different countries. It is evident that the proportion of annual publications output from China has experienced a substantial surge in recent years, while the proportion from the US has decreased slightly over time.

### 3.2 Authors and co-citation authors analysis

By examining the authors and their collaborative networks, we can gain insight into the cooperation between prominent researchers and key players in the field of breast cancer targeted therapy. This analysis will also aid in a better understanding of the research status and development trends in this area.

According to the calculation formula of core author proposed by Price, $Mp=0.749\sqrt{Npmax}$ , where *Mp* refers to the number of papers and *Npmax* represents the number of papers by the most published author (11). When an author’s number of published papers exceeds the core author limit (*Mp*), they are considered as a core author in the field. If the total number of papers published by core authors reaches 50% of all papers, it indicates that a core author group has formed in this field.

**Figure 3:**
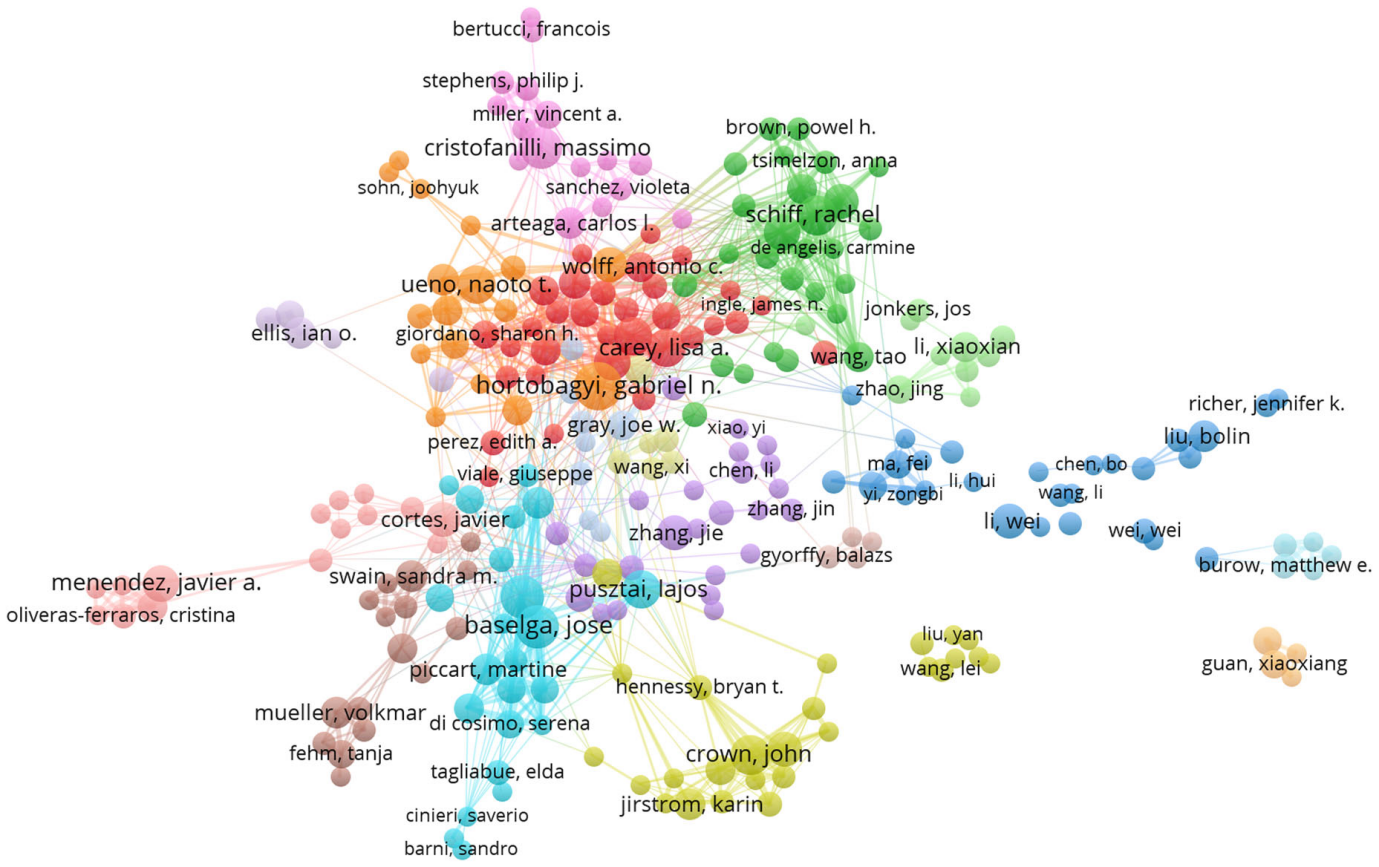
Author Collaboration Networks (N>3).The size of each node represents the weight of the core authors, the distance between nodes represents the degree of correlation between core authors, and the same color represents the same cluster. N is the number of publications by the author.

In this article, authors who have published more than three articles are considered core authors. A total of 336 core authors were identified, with a total of 1,805 articles, representing 79.93% of the total number of articles, which was in accordance with Price’s law. **Table 1** showed that Gabriel N. Hortobagyi had the highest number of published articles (N = 18), followed by José Baselga (N = 15) and Nadia Harbeck (N = 15). VOSviewer was run to set the minimum publication frequency threshold as 4, and the author cooperation network was generated, including 265 nodes and 1,326 links, which formed 17 clusters. C Kent Osborne had the highest total link strength (12), followed by Eric P Winer (13), Susan G Hilsenbeck (14), Gabriel N. Hortobagyi (14), and Rachel Schiff (15). We discovered that authors from the same cluster had close research collaborations (**Figure 3**). The top 10 most prolific authors also had notable collaborations, such as the connections between Massimo Cristofanilli, Naoto T Ueno, Lajos Pusztai, Francisco J Esteva, Lisa A Carey, and Gabriel N. Hortobagyi; between Lisa A Carey, Rachel Schiff, and Francisco J Esteva; and the partnership between José Baselga Baselga, Nadia Harbeck, and Lajos Pusztai. John Crown, however, represented a relatively independent research group. A total of 44,213 authors were found to have co-cited each other, with 12 authors having more than 200 co-citations. Dennis J Slamon ranked first with 653 co-citations, indicating that his literature has made significant contributions to the field. He was followed by José Baselga Baselga (583 citations) and Brian D Lehmann (277 citations)(**Table 1**). We also found that two authors, José Baselga and Lisa A Carey, were both listed in the top ten authors and co-cited authors. This indicates the significant impact these two authors have had on the field of targeted therapy for breast cancer.

**Table 1:** Top 10 authors and co-cited authors on breast cancer targeted therapy.

Rank	Author	Documents	Co-cited Author	Co-citation
1	Gabriel N. Hortobagyi	18	Dennis J Slamon	653
2	José Baselga	15	José Baselga	583
3	Nadia Harbeck	15	Brian D Lehmann	277
4	Massimo Cristofanilli	14	Luca Gianni	253
5	Rachel Schiff	13	Therese Sørlie	246
6	John Crown	13	Gunter von Minckwitz	240
7	Lisa A Carey	12	Charles M. Perou	235
8	Francisco J Esteva	12	Lisa A Carey	226
9	Lajos Pusztai	12	Rita Nahta	221
10	Naoto T Ueno	12	Daniel C Koboldt	220

### 3.3 Countries and institutions analysis

**Figure 4:**
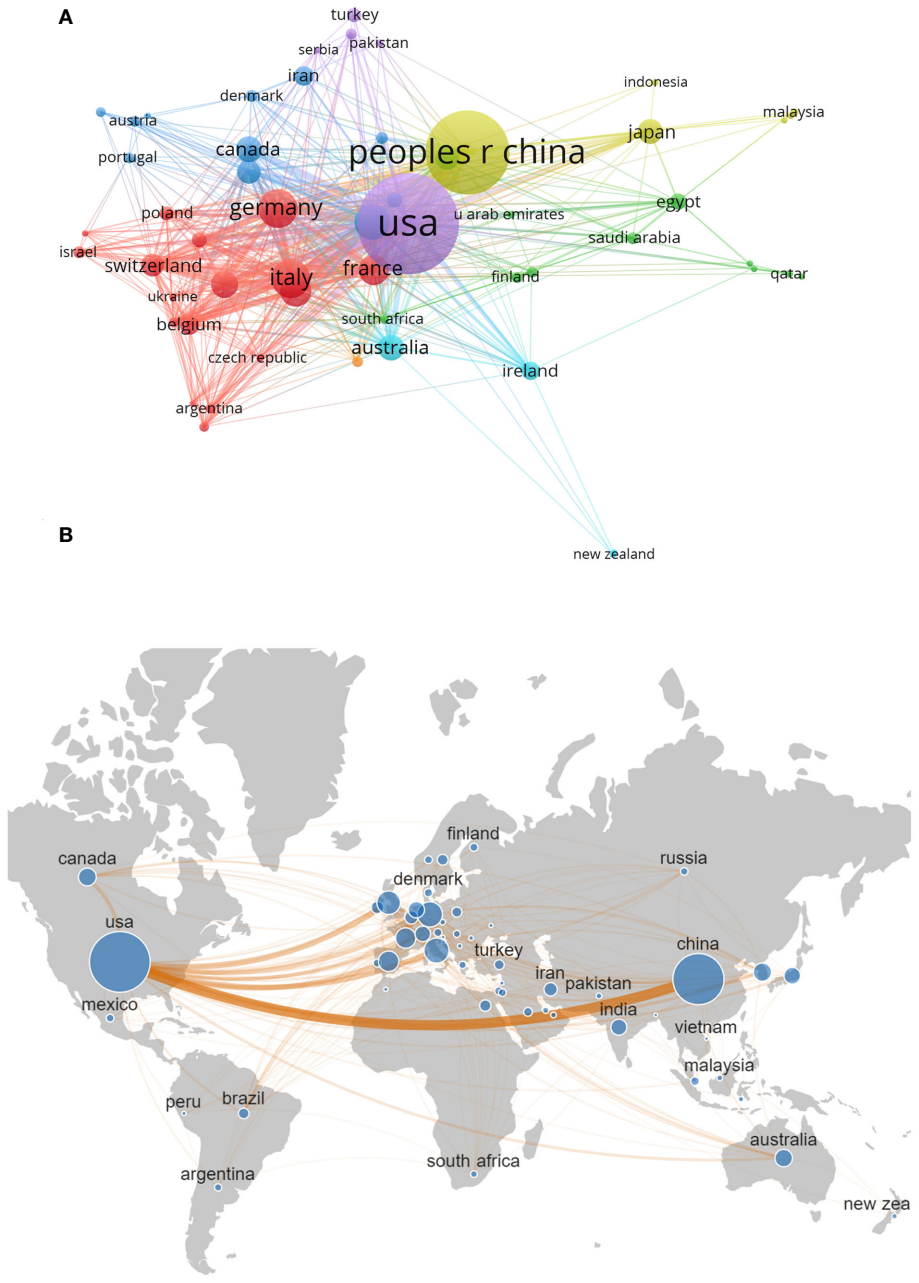
**(A)** Country Collaboration Network visualization (N≥3). The size of each node represents the number of articles published in each country, the line between the nodes represents the cooperation between the countries, and the same color represents the same cluster. N is the number of publications by country. **(B)** The network map of collaboration between countries.

A total of 85 countries worldwide conducted research related to targeted therapy of breast cancer between 2003 and 2022. Among them, the United States had the largest number of published articles (898 articles, 39.8%).China was also in the leading position (640 articles, 28.3%).Other countries with more than 100 articles published included Germany (156 articles, 6.9%), Italy (154 articles, 6.8%), the United Kingdom (135 articles, 6.0%), Spain (102 articles, 4.5%), and France (101 articles, 4.5%)(**Table 2**). In addition to the number of publications, the number of citations of a country’s published literature was also an important factor in assessing its impact in the field. The United States remained the country with the highest number of citations (45,312 citations), followed by China (14,276 citations), the United Kingdom (11,005 citations), Germany (9,188 citations) and Italy (6,905 citations).we selected 55 countries by setting the minimum publication threshold 3 to conduct the country collaboration network analysis. It was obvious that the United States was the central country in this field, with close cooperation with China, Germany, Italy, the United Kingdom, and Spain. The total link strength of the United States (703) is significantly higher than that of other countries (**Figure 4**).

**Table 2:** Top 10 productive countries on breast cancer targeted therapy.

Rank	Country	Documents	Citations	Average Citation
1	USA	898	45312	50.46
2	Peoples R China	640	14276	22.31
3	Germany	156	9188	58.90
4	Italy	154	6905	44.84
5	UK	135	11005	81.52
6	Spain	102	6615	64.85
7	France	101	4916	48.67
8	South Korea	81	3767	46.51
9	Canada	77	3460	44.94
10	Australia	74	4566	61.70

**Figure 5:**
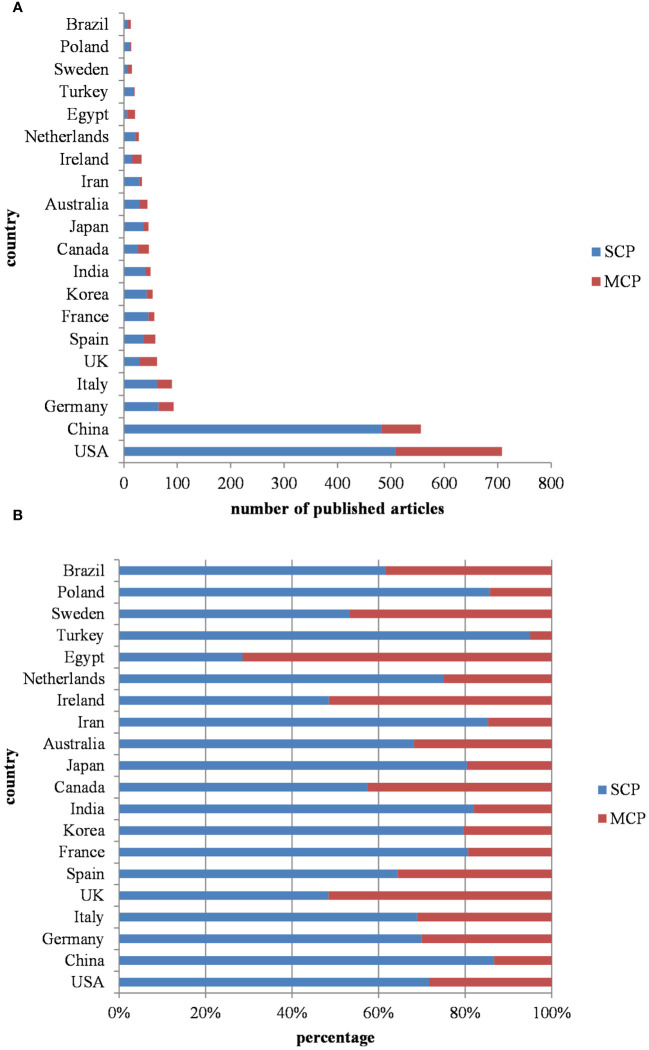
**(A)** Countries in the number of publications according to the country of corresponding authors. (Top 20) **(B)** Percentage of domestic and international cooperation in different countries. MCP: (multiple country publications), SCP (single country publications).

**Figure 5** illustrates the nationality distribution of corresponding authors and the extent of international collaboration. MCP (multiple country publications) denotes the number of papers co-authored with individuals from foreign countries, while SCP (single country publications) signifies the number of papers co-authored domestically. The MCP ratio, which is calculated as the proportion of papers co-authored internationally to the total number of papers published by authors of a particular nationality, offers insights into the degree of international cooperation within that group. The average MCP ratio for the top 20 countries in terms of publication volume is 0.252, indicating a predominantly domestic nature of their research collaborations. Turkey, China, and Poland display lower levels of international cooperation, as evidenced by their respective MCP ratios of 0.05, 0.133, and 0.143. This disparity may be attributed to factors such as geographical location, cultural background, and language barriers. In contrast, countries like Egypt, UK, and Ireland primarily disseminate research findings through international cooperation, with MCP ratios of 0.714, 0.516, and 0.515, respectively. This suggests a higher level of engagement in international collaboration.

**Figure 6:**
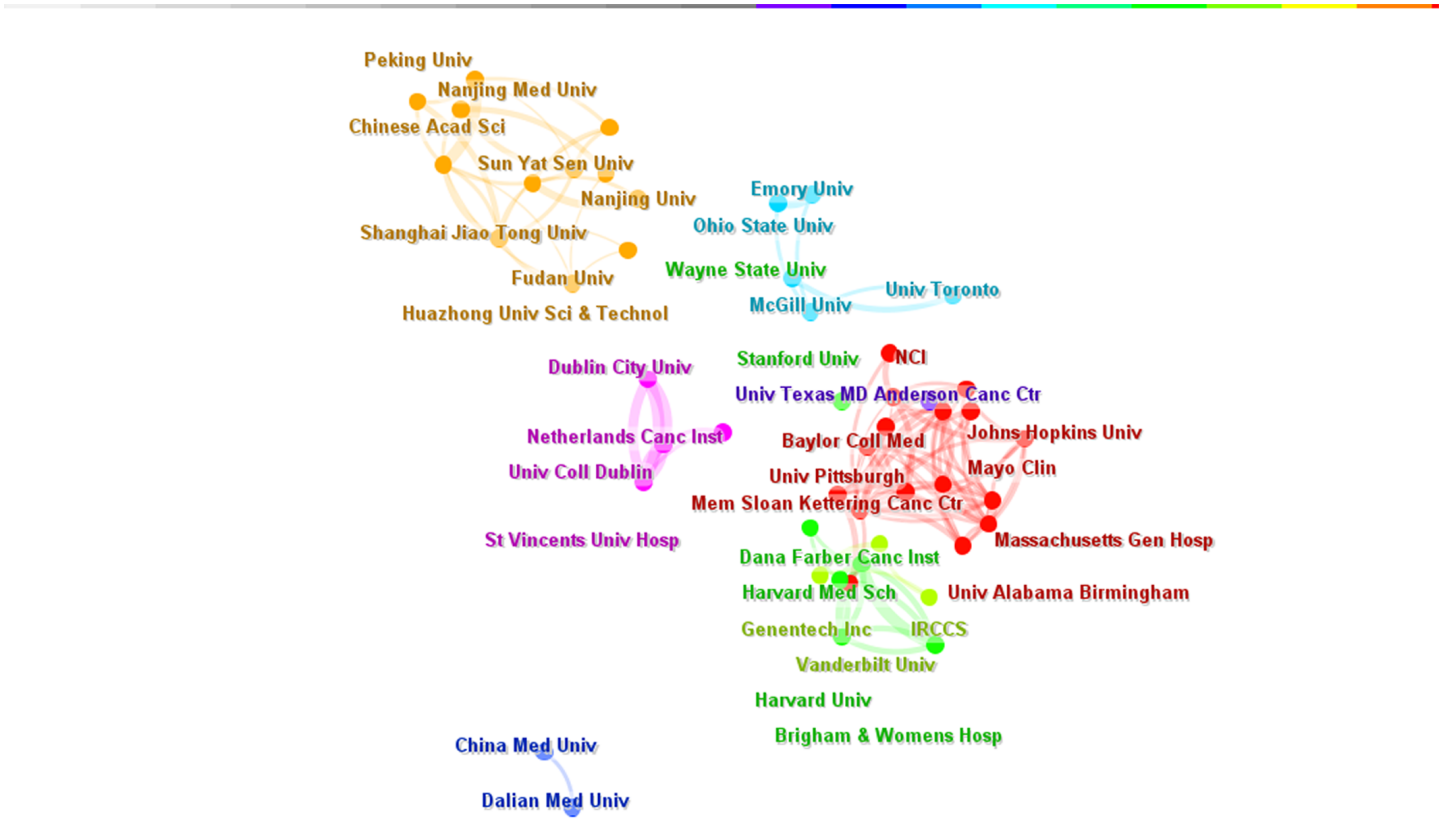
Institution Collaboration Network (N>3).The size of each node represents the weight of the institutions, the lines between nodes represent the cooperative relationship between institutions, and the same color represents the same cluster. N is the number of publications by institution.

A total of 3,230 institutions worldwide conducted research on targeted therapy for breast cancer. The top five institutions with the highest number of articles published in this field were the University of Texas MD Anderson Cancer Center (N=93), Memorial Sloan Kettering Cancer Center (N=66), Baylor College of Medicine (N=46), Dana-Farber Cancer Institute (N=45), and Fudan University (N=44)(**Table 3**). The top 10 institutions were from the United States (7/10) and China (3/10). The top-ranked institutions were all in the United States, and the citations of American institutions were significantly higher than those of Chinese institutions. The University of Texas M.D Anderson Cancer Center had the highest number of citations with 7,519, which was far surpassing other institutions. This demonstrated its leadership position in the field of oncology. We have selected 100 institutions (with a minimum number of 10 articles) for the analysis of institutional collaboration networks. The network contained 100 nodes and 785 links, resulting in 7 clusters (**Figure 6**). There was a close cooperative relationship between internal institutions within the country, especially the Memorial Sloan Kettering Cancer Center and the Dana Faber Cancer Institute, while cooperation between institutions from different countries is relatively limited.

**Table 3:** Top 10 institutions on breast cancer targeted therapy.

Institution	Publication	Total citations	Total link strength	Country
University of Texas MD Anderson Cancer Center	93	7519	259	USA
Memorial Sloan Kettering Cancer Center	66	5967	390	USA
Baylor College of Medicine	46	2544	141	USA
Dana-Farber Cancer Institute	45	5009	358	USA
Fudan University	44	830	51	China
Sun Yat-sen University	43	783	82	China
Harvard Medical School	37	1202	188	USA
Chinese Academy of Sciences	32	887	41	China
Vanderbilt University	31	1490	98	USA
Mayo clinic	29	2525	157	USA

### 3.4 Journals and co-cited journals analysis

A vast collection of 2,258 articles linked to breast cancer targeted therapy were published across 559 diverse journals. We listed the top 10 journals with the most published articles in this field (**Table 4**). Among these, Breast Cancer Research and Treatment (N=95, 4.21%) issued the most articles, Cancer Research contributed the most citations (5,711), and Clinical Cancer Research had the highest impact factor of 11.5. Among the top 10 journals, 6 were located in the United States, 3 in the United Kingdom, and 1 in Switzerland (**Table 4**). A remarkable 24.05% of all articles (N = 543) were published in the top 10 journals.

**Table 4:** Top 10 journals on breast cancer targeted therapy by publication numbers.

Rank	Source	Area	Documents	Citations	IF(2022)
1	Breast Cancer Research and Treatment	USA	95	3170	3.8
2	Oncotarget	USA	73	2636	5.168 (2016)
3	Clinical Cancer Research	USA	62	5304	11.5
4	Breast Cancer Research	UK	56	2315	7.4
5	Cancers	Switzerland	50	376	5.2
6	BMC Cancer	UK	45	1292	3.8
7	Scientific Reports	UK	43	966	4.6
8	Plos One	USA	41	1592	3.7
9	Cancer Research	USA	40	5711	11.2
10	Molecular Cancer Therapeutics	USA	38	2018	5.7

Among the 5,530 co-cited journals, there were 10 journals with co-citation counts exceeding 1,700 times (**Table 5**). The Journal of Clinical Oncology was the most co-cited journal, with a staggering N=5,684. Meanwhile, the New England Journal of Medicine stood out for its impact factor of 158.5 (**Table 5**). It was noteworthy to note that out of the top 10 journals, seven were published in the United States and three were from the United Kingdom.

**Table 5:** Top 10 co-cited journals on breast cancer targeted therapy.

Rank	Co-cited Journals	Area	Co-citations	IF (2022)
1	Journal of Clinical Oncology	USA	5684	45.4
2	Cancer Research	USA	4775	11.2
3	Clinical Cancer Research	USA	3759	11.5
4	New England Journal of Medicine	USA	2540	158.5
5	Breast Cancer Research and Treatment	USA	2470	3.8
6	Oncogene	UK	2256	8.0
7	Proceedings of the National Academy of Sciences of the United States of America	USA	2255	11.1
8	Nature	UK	2232	64.8
9	Annals of Oncology	UK	1911	50.5
10	Journal of Biological Chemistry	USA	1707	4.8

### 3.5 Co-cited references analysis

We employed VOSviewer to scrutinize the top 10 co-cited references, and all of them had a co citation frequency of at least 100 times. These top 10 co-cited references were classified as representative articles of three distinct theme clusters (**Table 6**). Cluster 1#: Identification of molecular subtypes and clinical features of breast cancer. Cluster 2#: The role of HER2 gene in breast cancer and the effect of anti-HER2/EGFR-TKI drugs in the treatment of HER2-positive breast cancer. Cluster 3#: Clinical trials related to the efficacy of trastuzumab. We put the references in chronological order to analyze so as to better understand how ideas and knowledge have developed over time, and to better identify trends in the future.

**Table 6:** Top 10 co-cited references on breast cancer targeted therapy.

Rank	Title	Author	Journal	Citations	Cluster
1	Use of chemotherapy plus a monoclonal antibody against HER2 for metastatic breast cancer that overexpresses HER2.	Dennis J. Slamon(2001) (12)	New England Journal of Medicine	262	3
2	Human Breast Cancer: Correlation of Relapse and Survival with Amplification of the HER2/neu Oncogene	Dennis J. Slamon(1987) (13)	Science	247	2
3	Comprehensive molecular portraits of human breast tumors	Daniel C. Koboldt (14)	Naure	216	1
4	Molecular portraits of human breast tumors	Charles M. Perou(2000) (4)	Naure	210	1
5	Identification of human triple-negative breast cancer subtypes and preclinical models for selection of targeted therapies	Brian D Lehmann(2011) (15)	Journal of Clinical Investigation	196	1
6	Triple-negative breast cancer: clinical features and patterns of recurrence	Rebecca Dent(2007) (16)	Clinical Cancer Research	173	1
7	Gene expression patterns of breast carcinomas distinguish tumor subclasses with clinical implications	T Sørlie(2001) (17)	Proceedings of The National Academy of Sciences of The United States of America	149	1
8	Trastuzumab plus adjuvant chemotherapy for operable HER2-positive breast cancer	Edward H Romond(2005) (18)	New England Journal of Medicine	135	3
9	Triple-negative breast cancer	William D Foulkes(2010) (19)	New England Journal of Medicine	134	1
10	Lapatinib plus capecitabine for HER2-positive advanced breast cancer	Charles E Geyer(2006) (20)	New England Journal of Medicine	132	2

In 1987, Slamon et al. published an article in Science titled “Human Breast Cancer: Correlation of Relapse and Survival with Amplification of the HER-2/neu Oncogene.” This groundbreaking study first revealed that the amplification of the HER-2/neu oncogene was an important predictor of the overall survival and time to relapse for breast cancer patients. The authors argued that HER2, as a driving gene, played a significant role in the biological behavior and pathogenesis of human breast cancer (13).

In 2000, Perou et al. published an article in Nature titled “Molecular portraits of human breast tumors.” This seminal work ushered in a new era of breast cancer classification by leveraging gene expression patterns. By employing cutting-edge molecular analysis techniques, the traditional morphology-based system was replaced with a novel one that relied on molecular features. As a result, breast cancer was subdivided into four distinct categories: ER+/luminal-like, basal-like, Erb-B2+, and normal breast (4). Subsequently, in 2001, Sorlie et al. further classified ER+/luminal-like tumors into type A and type B/C (17). The addition of type A and type B/C helped to distinguish between different subgroups of ER+/luminal-like tumors, which can have distinct clinical features and response to treatment.

In the same year, Slamon et al. published the “Use of chemotherapy plus a monoclonal antibody against HER2 for metastatic breast cancer that overexpress HER2” in the New England Journal of Medicine. This study demonstrated the effectiveness of a combination therapy consisting of trastuzumab and first-line chemotherapy for metastatic breast cancer with HER2 overexpression. The results of large clinical trials published in the article showed that this combination treatment significantly improved outcomes compared to standard chemotherapy alone, and had a profound impact on the treatment of metastatic breast cancer (12).

In 2005, the article published by Romond et al. in the New England Journal of Medicine reported the results of the NSABP B-31 study, which showed that after a regimen of doxorubicin and cyclophosphamide, adding trastuzumab to paclitaxel reduced recurrence by 50% among women with HER2-positive breast cancer and reduced distant recurrence by 8.8% at 3 years (18).

In 2006, Geyer et al. unveiled the findings of a phase III randomized open label study, revealing that for HER2-positive breast cancer patients who encountered a recurrence after undergoing a regimen involving anthracyclines, taxanes, and trastuzumab, the combination of lapatinib and capecitabine significantly prolonged the median progression time while reducing the risk of disease progression by 51% when compared to capecitabine alone (20).

The article published by Dent et al. in Clinical Cancer Research in 2007 presented a large-scale study conducted at a single institution with long-term follow-up on triple-negative breast cancer (TNBC) patients. The article detailed the clinical characteristics and recurrence patterns of this subtype, highlighting the heightened risk of early distant recurrence and mortality (16).

In 2010, Foulkes et al. published their comprehensive review in New England Journal of Medicine. The article delved into the origins, molecular and clinical features, as well as treatment options for TNBC (19).

In 2011, Lehmann et al. published their study in the Journal of Clinical Investigation, which utilized gene expression profiling and cluster analysis to identify six distinct subtypes of TNBC. The researchers found that these subtypes exhibited differences in recurrence-free survival and drug sensitivity, highlighting the need for targeted therapies tailored to each subtype. This discovery has significant implications for the development of diagnostic biomarkers and TNBC-specific drugs, as understanding the underlying molecular mechanisms of TNBC can lead to more effective treatments for this challenging and understudied form of breast cancer (15).

In 2012, Koboldt et al. used six different techniques to analyze genetic and epigenetic abnormalities in tumor tissues from patients with breast cancer. This study represented a significant breakthrough in the understanding of breast cancer, as it allowed for a comprehensive analysis of the molecular subtyping of this disease. The researchers utilized cutting-edge technologies, including high-throughput sequencing and multi-omics techniques, to identify unique genetic and molecular markers in each subtype of breast cancer. By confirming the existence of four main subtypes of breast cancer, this study opened the door for more targeted therapies and personalized medicine approaches to help improve patient outcomes. This research also had the potential to lead to advancements in early detection and prevention strategies, as well as improved treatment options for those diagnosed with breast cancer (14).

### 3.6 Keywords analysis

Keywords summarize the theme of a paper. By exploring keywords co-occurrence and clustering, we can investigate the hotspots topics and development trends in the research field. We used the VOS viewer to analyze the frequency of keywords of the 2,258 publications. After merging synonymous keywords, we were able to identify a total of 7,527 unique keywords. The details of the top 20 frequencies are listed in **Table 7**.

**Table 7:** Top 20 high-frequency keywords related to breast cancer targeted therapy research from 2003 to 2022.

Rank	Keyword	Occurrences	Year
1	breast cancer	787	2003
2	expression	610	2003
3	triple negative breast cancer	397	2009
4	targeted therapy	327	2005
5	trastuzumab	306	2005
6	therapy	273	2004
7	HER2	272	2006
8	resistance	252	2006
9	metastasis	249	2005
10	chemotherapy	248	2004
11	survival	248	2005
12	cells	246	2003
13	growth	236	2004
14	activation	221	2004
15	apoptosis	185	2009
16	pathway	168	2008
17	receptor	159	2005
18	phase-ii trial	157	2004
19	carcinoma	155	2003
20	gene/lapatinib	142	2004

**Figure 7:**
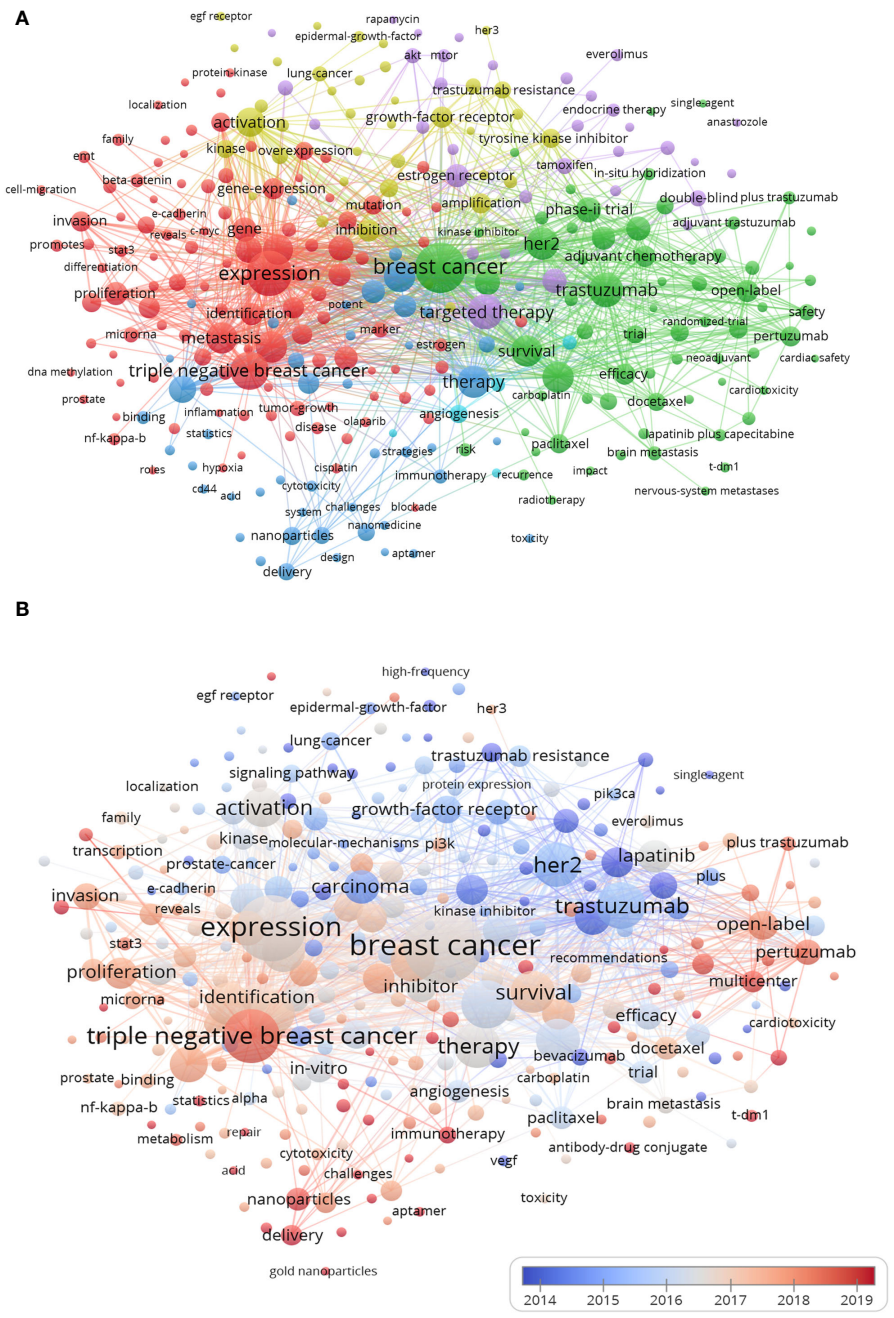
Keywords co-occurrence network and cluster map (N>10). **(A)** Network visualization. The size of each node indicates the co-occurrence frequency of the corresponding keyword, and the larger the nodes and fonts, the higher the frequency of occurrence. The thickness of the connecting lines between nodes indicated how many times they co-occur, the same color indicates the same cluster, and N is the co-occurrence frequency of keywords. **(B)** Overlay visualization. Overlay visualizations depict the hotspots in the field within breast cancer targeted therapy. The VOSviewer tool employs a color-coding system to represent keywords according to the specific year of their occurrence in the literature.

The minimum occurrence frequency threshold of keywords was set as 11, and a total of 307 nodes were obtained after synonym merging to generate keywords co-occurrence and clustering network map, as shown in **Figure 7A**. The keywords were divided into 6 clusters.

The cluster 1 has 113 keywords, including: expression, TNBC, cells, growth, metastasis, carcinoma, prognosis, proliferation, progression, and etc. This cluster provided valuable insights into the mechanisms of biological behavior regulation of breast cancer, as well as the potential targets for targeted therapy in TNBC patients. The cluster 2 comprised a total of 68 keywords, encompassing breast cancer, trastuzumab, HER2, chemotherapy, survival, pertuzumab, lapatinib, phase-II trial, open-label, efficacy, and etc. This cluster primarily focused on investigating the safety and efficacy of breast cancer chemotherapy as well as targeted HER-2 therapies through clinical trials. The cluster 3 contained 47 keywords, referring to: therapy, resistance, mechanism, inhibitor, apoptosis, nanoparticles, *in-vitro*, etc. This cluster mainly related to breast cancer targeted inhibitor resistance and cell apoptosis signaling pathways, as well as the development of targeted drug delivery systems *in vitro* and *in vivo*. The cluster 4 included 39 items such as activation, inhibition, amplification, epidermal growth factor receptor (EGFR), kinase, growth factor receptor, tyrosine kinase inhibitor, drug resistance, etc. It mainly related to regulation of EGFR family signaling pathways and mechanisms related to drug resistance of tyrosine kinase inhibitors. The cluster 5 focused on the breast endocrine therapy had 35 items: targeted therapy, combination, estrogen receptor, tamoxifen, etc. The last cluster included only 5 items, which was about antiangiogenic therapy in breast cancer. Cluster 2 had the highest link strength and co-occurrence frequency among the keywords, while cluster 3 had the latest publication time. These findings indicate that cluster 2 represents a focused area of research in breast cancer targeting, whereas cluster 3 signifies an emerging field of study.

The overlay visualization map (**Figure 7B**) demonstrated the hotspots in this field. The VOSviewer used a color-coding system to differentiate between keywords based on their year of appearance in literature. The earlier keywords appeared in blue, followed by red colors for later years. Some of the most recent keywords included TNBC, nanoparticles, delivery, pertuzumab, multicenter, T-DM1 and immunotherapy.

### 3.7 Keywords burst analysis

**Figure 8:**
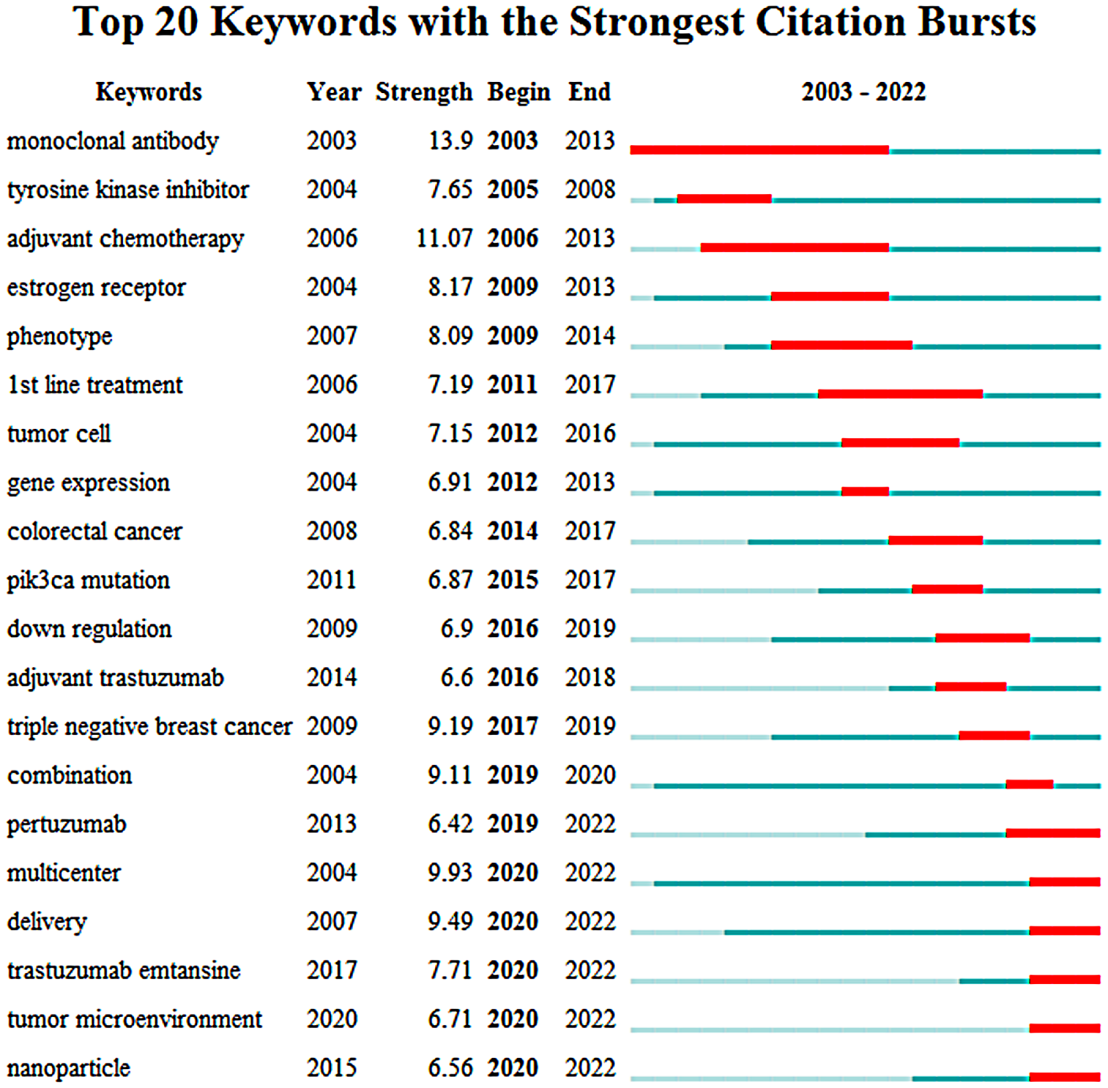
The evolution trend of burst words on targeted therapy for breast cancer from 2003 to 2022.

Keywords burst analysis is a technique that can be used to identify the evolution of research hotspots over time and predict future research trends. In this study, we analyzed a total of 2,258 articles from 2003 to 2022 using the burst detection algorithm of CiteSpace. The top 20 keywords with the strongest bursts were identified and presented in **Figure 8**. The keywords, earliest year in which the keywords appeared, burst strengths, and the starting and ending years of the keywords years were also presented in **Figure 8**. The red strip at a specific location in the blue timeline indicated the time interval during which the burst of a keyword lasted.

Before the year 2013, research in this area was largely focused on identifying and developing new drugs that could target specific proteins or pathways involved in breast cancer development and progression. This led to the development and evaluation of drugs like trastuzumab, which targeted the HER2 protein in breast cancer cells, and lapatinib, which was a tyrosine kinase inhibitor that could target both HER2 and EGFR proteins. In addition, research was conducted on the expression and phenotype of genes related to therapeutic targets in tumor cells. As research in targeted therapy continued to advance, researchers began to focus more on understanding the underlying genetic and molecular mechanisms of biological behaviors in breast cancer. This led to the discovery of new genes and pathways that may be targeted by drugs, as well as new approaches to analyzing tumor cells and identifying potential therapeutic targets.

Since 2013, pertuzumab, a new anti HER2 drug, had gradually become the focus in the field of molecular targeted therapy for breast cancer. At the same time, multi-center clinical trials had explored the efficacy and safety of trastuzumab combined with pertuzumab or cytotoxic drugs in the treatment of HER2 positive breast cancer. In addition to this, there had been significant progress in understanding the molecular mechanisms underlying the development and progression of TNBC, as well as in developing new drugs for patients with PIK3CA-mutated breast cancer. Finally, there had also been a growing interest in exploring the use of nanoparticle drug delivery agents as a way to improve the efficacy and safety of targeted therapies for breast cancer, which is an exciting area of research that holds great promise for the future of breast cancer treatment.

It is worth noting that the keywords “pertuzumab”, “multicenter”, “delivery”, “trastuzumab emtansine”, “tumor microenvironment” and “nanoparticles” are still in the outbreak stage and represent the current frontier hot spots and future trends of molecular targeted therapy for breast cancer. This indicates that these specific keywords have been identified as the current forefront of molecular targeted therapy for breast cancer and may continue to be important areas of research in the future.

## 4 Discussion

We conducted a bibliometric analysis of 2,258 publications from 85 countries and found that the number of publications in the field of targeted therapy for breast cancer has shown a steady increase year by year over the past two decades. In recent years (2018–2022), the number of publications accounted for nearly half of the total, indicating that this field has become the focus of attention. From the analysis of national network distribution, the United States ranked first in the number of publications, accounting for 39.8% of the global number of publications, showing the core position of the United States in the research of targeted therapy for breast cancer. This may be related to the USA’s investment in cancer research. In a study published in The Lancet, a research team collected investment data on public and charitable cancer research globally from 2016 to 2020, totaling $24.5 billion in funding for a total of 66,388 cancer research projects. By country, the United States provided 57.3% of the total funding for cancer research, with the United Kingdom accounting for 9.8%, the European Union for 5.4%, and China for 4.4% (21). It is surprising that among the top 10 countries in the number of publications, China, as the only developing country, ranks second with 640 publications, indicating a huge investment in breast cancer targeted therapy research and efficient use of funding. Overall, the combination of increased research funding, international collaborations, and improved research infrastructure has propelled China to become a major participant in breast cancer targeted research, reflected by the significant rise in numbers and proportion of annual publications from China over the past two decades. However, when considering an alternative viewpoint, it is noteworthy that despite China’s second-place ranking in terms of overall publication volume, it lags behind in terms of average citation frequency for articles. This suggests that there is room for improvement in the research quality and impact of Chinese researchers.

Among the top 10 institutions in terms of publication volume, the University of Texas MD Anderson Cancer Center ranks first, indicating that it possesses outstanding research strength and influence in the field of breast cancer targeted therapy. The fact that seven of these institutions are located in the US and only three are in China also highlights the significant advantage that the US has in terms of publishing volume and research influence. Meanwhile, as a developing country, China is striving to catch up with the research in breast cancer targeted therapy.

From the analysis of authors and co-cited authors, the core author group on targeted therapy for breast cancer has been formed. Gabriel N. Hortobagyi is the author with the highest number of publications (N=18), and Dennis J Slamon is the author with the highest number of co-citations (N=653). The pioneering work of renowned academic Dennis J Slamon helped to develop the breakthrough cancer drug trastuzumab, which has saved and prolonged the lives of millions of patients (13, 22). Dr. Slamon is also a leader in the development of CDK4/6 inhibitors for breast cancer therapy, which paves the way for new strategies to treat this devastating disease (23). In addition, we found that José Baselga and Lisa A Carey are both in the top 10 authors and the top 10 co-cited authors, which reflects their authority and dominance in this field.

From the collaboration between institutions and authors, we found that there exists an obvious national boundary in the cooperative network, implying that the degree of collaboration and communication between institutions and authors in different countries is relatively low. Within countries, academic exchanges are mainly organized around a few important institutions to form a number of cooperative groups, which reflects the close collaboration between domestic institutions and authors. However, at the international level, such cooperation needs to be further improved. A global analysis of cancer research funding predicts that approximately 75% of cancer deaths will occur in low- and middle-income countries by 2030. However, cancer research is heavily skewed toward high-income countries, with only 0.5% of investment allocated to cancer research in low- and middle-income countries in this analysis (21). In order to promote the continuous development of targeted therapy for breast cancer, we should encourage and strengthen international cooperation and communication, enabling institutions and authors from different countries and regions to participate in collaborative research in this field.

According to statistics, among the 2,258 published papers included in the statistics, Breast Cancer Research and Treatment was the major source of published papers, but it did not have a significant advantage in impact factor and citation. This phenomenon may reflect a certain gap in the research content and quality published in this journal compared to other high-level journals. We found that the United States and the United Kingdom had an unrivalled dominant position in the academic research field of breast cancer after we analyzed the geographical distribution of the publishing regions of the top 10 journals and the top 10 cited journals. This phenomenon highlights the dominant position of Europe and the United States in the academic research of breast cancer, and also reflects the global researchers tend to choose those well-known journals located in Western countries when publishing their research results.

Among the top 10 highly co-cited references, there were 5 basic researches, 3 clinical trials, 1 clinical research and 1 review. These articles were all published in the top journals which were classified as Q1 by the 2022 journal citation report. From the perspective of the timeline, these highly cited articles well demonstrate the development process and trend of targeted therapy for breast cancer. Early research mainly focused on molecular subtype of breast cancer, HER2 target basic research and clinical application of trastuzumab (4, 12, 13, 17, 18, 20). Over time, the focus has gradually shifted to the clinical characteristics, recurrence patterns, molecular genetic drivers and targeted drug development of TNBC (15, 16, 19). This trend reflects the continuous advancements and in-depth exploration of breast cancer targeted therapy, providing researchers with valuable insights into future development directions. These articles also demonstrate the immense potential of translational medicine in translating basic research findings into clinical treatments through rigorous clinical trials, ultimately benefiting those in need. Therefore, for researchers focused on breast cancer targeted therapy, a comprehensive analysis of the research accomplishments and trends presented in these highly cited publications can aid in further breakthroughs and advancements within this field.

Keyword analysis reveals that in the field of targeted therapy for breast cancer, there is a two-pronged approach to research, comprising both clinical application research and basic research. Clinical application research primarily focuses on evaluating the efficacy and safety of new targets, drugs, and combination therapies through clinical trials (24–26). For example, the NSABP B-31/-N9831 study established that AC-TH (doxorubicin combined with cyclophosphamide sequential paclitaxel drugs combined with trastuzumab) is superior to conventional AC-T chemotherapeutic agents for early-stage HER2-positive breast cancer (27). The APHINITY study found that the dual targeted treatment regimen containing both trastuzumab and pertuzumab was more effective in reducing the risk of recurrence than the regimen containing trastuzumab alone, especially in lymph node positive patients (28). The phase III clinical trial (NCT01958021) and (NCT02278120) demonstrated that ribociclib and letrozole combination therapy can improve the progression-free survival (PFS) of postmenopausal and premenopausal women with HR+/HER2-negative breast cancer, respectively (29, 30). Basic research, on the other hand, encompasses studies on the classification of breast cancer, regulation mechanisms of biological behaviors such as proliferation, angiogenesis, invasion, metastasis, drug resistance, and apoptosis in breast cancer cells (31–34). The six TNBC classification scheme proposed by Lehmann et al. has been verified by several clinical trials (15). Fudan classification uses Asian population samples for research and plays a more targeted guiding role in precision medicine in China (31). Song-Yang Wu et al. found that CD8-positive T cells could identify an immunomodulatory subpopulation of TNBCs with a higher possibility to benefit from immunotherapy. Patients with somatic polycystin-1 mutations showed poor response to therapy. The combined detection of CD8, programmed cell death-ligand 1(PD-L1), and somatic gene mutations could accurately distinguish TNBC patients who could benefit from immunotherapy (35). Anna Adam-Artigues et al. identified receptor tyrosine kinase AXL overexpression as an essential mechanism of trastuzumab resistance and found that AXL could be used as a targetable prognostic biomarker in HER2-positive breast cancer (36). Additionally, it involves researching the development of new target drugs, and drug delivery vectors based on cell signaling pathways (37). For example, *in vitro*, lipid-modulating agent simvastatin was encapsulated within trastuzumab-functionalized liposomes for targeting HER2-positive breast cancer cells (38). Kai Xiao et al. reported a luteinizing hormone-releasing hormone (LHRH) receptor-targeted and tumor microenvironment-responsive nanoparticle system to selectively deliver chemotherapeutic drugs to TNBC cells, with promising results demonstrated *in vivo* (39).

Keyword burst analysis found that research on targeted drugs for breast cancer mainly focused on HER2 and ER/PR (6). In the past 20 years, drug therapy has shown a research trend from single target to multi-targets and from single drug to combination therapy (40, 41). With the continuous improvement of molecular subtype classification of breast cancer, anti-HER2 and anti-ER/PR drugs have achieved good therapeutic effects as targeted drugs for breast cancer. However, drug resistance reduces its therapeutic effect. For example, the activation of the PI3K/Protein kinase B(Akt)/mammalian target of rapamycin (mTOR) pathway can lead to estrogen-independent activation of ER and has been associated with resistance to endocrine therapy (42). Activation of this pathway is also implicated in acquired resistance to HER2-targeted therapy (43, 44). Several mechanisms of resistance to HER2-targeted therapy mainly include gene mutations leading to protein conformational changes affecting antigen-antibody binding ability, abnormal activation of downstream and bypass signaling pathways, and the failure of the host immune system to mount an appropriate response against HER2-positive cancer cells, etc. (34, 40, 45). Thus, research on the regulatory mechanisms of drug resistance, such as gene mutation, hormonal regulation, cell cycle and apoptosis regulation, epigenetic changes and tumor microenvironment, have emerged as another important trends in the field of targeted therapy for breast cancer over the past 20 years (8, 46).

The emerging trends of targeted therapy for breast cancer have been constantly evolving. In the future, the trend of targeted therapy for breast cancer may include the following three aspects. First, the trend of targeted therapy for breast cancer may involve a multi-target and multi-drug combination therapy strategy. The characteristics of tumor cells include vascular proliferation, apoptosis inhibition, immune escape, among others. This suggests that treatment of tumors should be holistic and multi-faceted, involving multiple drugs targeting different aspects of the disease. Combination treatment regimens increase the effectiveness of therapy and provide a more efficient solution to breast cancer resistance compared to single-agent therapy. Additionally, combination therapies can help reduce the toxicity associated with some treatments, making them more tolerable for patients. Studies have shown that the combination of mTOR1 inhibitor everolimus and tamoxifen can significantly decrease the incidence of secondary drug resistance and intolerable side effects (47, 48). A randomized phase III trial found that the combination of exemestane and everolimus led to significant improvements in progression-free survival (PFS) compared to exemestane alone in patients with metastatic breast cancer (22). In recent years, antibody-drug conjugates (ADCs) targeting HER2 have gained significant attention as a promising treatment option for patients with HER2-positive breast cancer who have developed resistance to traditional HER2-targeted therapies. Trastuzumab emtansine (T-DM1), has been approved for patients with residual invasive lesions following neoadjuvant therapy (49). In addition, trastuzumab deruxtecan (T-Dxd) provides new therapeutic options for patients with advanced HER2-positive and HER2-low breast cancer and improves their chances of long-term survival (50–52). In addition to the examples mentioned, there are also ongoing studies exploring the potential of combination therapies involving targeted drugs and immunotherapies. For instance, researchers are investigating the efficiency and safety of atezolizumab(an anti–PD-L1 antibody) in combination with trastuzumab plus pertuzumab in HER2-positive, early high-risk and locally advanced breast cancer (53). Overall, the development of drugs that target multiple pathways and use combination strategies has great potential for improving the safety and efficacy in breast cancer targeted therapy.

Second, the exploration of drugs that specifically target novel signaling pathways and therapeutic targets in TNBC may be another direction for future research. TNBC is one of the most malignant subtypes of breast cancer with poor prognosis and highly molecular heterogeneity (54). Lehmann, Burstein and Yizhou Jiang et al. all proposed different classification schemes for TNBC subtypes, which reflects the complexity of TNBC subtype classification and the difficulty of developing new treatment options based on different subtypes, molecular characteristics and signaling pathways (15, 31, 55–57). Currently, the identification of subtypes and molecular features, and the development of more effective therapies based on these are active areas of research. Currently, a variety of targeted therapeutic strategies have emerged, including PARP inhibitors, antibody-drug conjugates (ADCs),PI3K/AKT/mTOR inhibitors, Notch inhibitors, and et al. At present, PARP inhibitors targeting breast cancer susceptibility gene (*BRCA*) mutations and trophoblast cell-surface antigen 2 (trop2) ADC drug sacituzumab govitecan (SG) have been used in clinical practice. The phase III OlympiAD (NCT02000622) study confirmed olaparib significantly prolonged the PFS of patients with HER2-negative metastatic breast cancer and a germline *BRCA* mutation (58). Based on the OlympiA trial (NCT02032823), 1 year of adjuvant olaparib is recommended for patients with high-risk germline *BRCA* mutation HER2-negative early breast cancer (59). One promising target for TNBC therapy is trop2.Trop2 ADC drugs sacituzumab govitecan (SG) have been approved and achieved excellent efficacy in mTNBC (60). Another clinical trials about Datopotamab deruxtecan(another trop2-ADC) plus durvalumab(anti-PD-L1) are also ongoing, which demonstrate manageable safety and compelling high, durable response rates in locally advanced/metastatic TNBC (61). Besides, immune checkpoint inhibitors may be another promising approach. Clinical trials are currently underway to evaluate the efficacy of immune checkpoint inhibitors in TNBC (62). Researchers are also exploring the role of other signaling pathways and molecular targets in TNBC, such as PI3K/AKT/mTOR, Notch, and Wnt/β-catenin pathways, which may represent potential therapeutic targets in the future (63, 64). These endeavors will undoubtedly offer more treatment options for TNBC and ultimately contribute to improved outcomes.

Furthermore, the utilization of nanotechnology in breast cancer treatment is a burgeoning area of research in recent years (65, 66). In nanomedicine for cancer treatment, a large number of nanomaterials have been used as carriers to deliver cytotoxic drugs to cancer tissue. Examples of nanomaterials used as carriers in cancer treatment include liposomes, solid lipid nanoparticles, nanostructured lipid carriers, polymeric nanoparticles, inorganic nanoparticles, hybrid nanoparticles, and et al (67). Nanoparticles (NPs) have emerged as a promising technology for targeted drug delivery in breast cancer treatment. By conjugating NPs with specific ligands, they can selectively accumulate in tumor tissues to increase the concentration of drugs at the tumor site and improve the therapeutic efficacy. This can result in a lower effective dose of the drug, reducing the risk of side effects and improving patient tolerability. Additionally, NPs can protect the encapsulated drugs from degradation and enhance pharmacokinetic properties, prolonging circulation time and increasing tumor uptake (68). Several studies have demonstrated the potential of NP-mediated drug delivery systems in breast cancer treatment. For example, tamoxifen-loaded solid lipid nanoparticles have been shown to overcome tamoxifen resistance by inducing apoptosis in breast cancer cells (69). Similarly, detachable immune liposomes loaded with paclitaxel and anti-CD47 antibody have been developed for targeted chemotherapy against TNBC, resulting in improved antitumor efficacy against TNBC and inhibited tumor recurrence (70). Many chemotherapy agents such as adriamycin, paclitaxel and cisplatin have been found to display increased targeted cytotoxic efficacy when delivered as part of a nanoparticle delivery systems (68, 71–73). However, there are currently limited data on the long-term safety and efficacy of NPs in patients, as most of the available studies have been conducted in preclinical models or small cohorts of patients, clinical translation for managing breast cancer remains slow. Further research is required in the near future to address these challenges and optimize the clinical translation of NPs for the treatment of breast cancer.

## 5 Limitation

Our study has certain limitations, mainly stemming from the fact that we only searched for English publications in a single WOS database, which may result in a degree of selection bias in our study, potentially leading to incompleteness in the included data. To improve the reliability and accuracy of our results, we should consider incorporating a wider range of databases and languages to facilitate comprehensive collection of related research. By doing so, we can increase the overall quality and usefulness of our analysis for researchers and practitioners in the field.

## 6 Conclusion

In this article, we summarized the development of targeted therapy research in breast cancer over a 20-year period through bibliometric analysis and identified potential research directions. The research in this area was mainly focused on development and clinical evaluation of agents associated with the EGFR family signaling pathway and tyrosine kinase, as well as pathogenesis, drug resistance and metastasis mechanisms of breast cancer. Research frontier in this research field included combination therapy strategy, TNBC, and nanotechnology. The annual number of papers published showed an upward trend and a core author group has formed in this field. The United States was the most prolific country and University of Texas MD Anderson Cancer Center contributed the largest number of publications. The authors emphasized the importance of fostering collaboration among researchers, countries, and institutions to advance the field.

## Data availability statement

The original contributions presented in the study are included in the article/supplementary material. Further inquiries can be directed to the corresponding author.

## Author contributions

DW: Conceptualization, Methodology, Visualization, Writing – original draft, Writing – review & editing. CP: Formal analysis, Visualization, Writing – original draft, Writing – review & editing. YH: Data curation, Visualization, Writing – review & editing. ZS: Data curation, Supervision, Writing – review & editing. YZ: Methodology, Supervision, Writing – review & editing. MX: Conceptualization, Writing – review & editing.
